# Fiber Connectivity Density in Cerebral Small-Vessel Disease Patients With Mild Cognitive Impairment and Cerebral Small-Vessel Disease Patients With Normal Cognition

**DOI:** 10.3389/fnins.2020.00083

**Published:** 2020-02-12

**Authors:** Chengxia Liu, Lin Shi, Wenhao Zhu, Shiqi Yang, Pan Sun, Yuanyuan Qin, Xiangyu Tang, Shun Zhang, Yihao Yao, Zhenxiong Wang, Wenzhen Zhu, Defeng Wang

**Affiliations:** ^1^Department of Radiology, Tongji Hospital, Tongji Medical College, Huazhong University of Science and Technology, Wuhan, China; ^2^Department of Medicine and Therapeutics, The Chinese University of Hong Kong, Hong Kong, China; ^3^Department of Neurology, Tongji Hospital, Tongji Medical College, Huazhong University of Science and Technology, Wuhan, China; ^4^Xianning Central Hospital, Xianning, China; ^5^Department of Imaging and Interventional Radiology, The Chinese University of Hong Kong, Hong Kong, China

**Keywords:** cerebral small-vessel disease, cognition, diffusion tensor imaging, white matter hyperintensity, structural connectivity

## Abstract

Abnormal structural connectivity of cerebral small-vessel disease (CSVD) is associated with cognitive impairment. But the different characteristics of structural connectivity have not been elucidated in early CSVD patients. The current study aimed to investigate the potential differences of structural connectivity in CSVD patients with mild cognitive impairment (MCI) and CSVD patients with normal cognition. Twenty-two CSVD patients with MCI, 34 CSVD patients with normal cognition, and 35 controls, who were age, sex, and education matched underwent diffusion tensor imaging and high resolution T1-weighted imaging. Clinical characteristics, lacunar infarct volume, white matter hyperintensity (WMH) volume, and global atrophy were quantitatively evaluated. Maps of fiber connectivity density (FiCD) were constructed and compared across groups in vertex levels. Pearson correlation was used to estimate the imaging–clinical relationships with control of general characteristics. CSVD patients with MCI had higher lesion load of WMH and lacunar infarcts, and correspondingly lower global FiCD value than CSVD patients with normal cognition (*P* < 0.01). Lacunar infarct (*r* = −0.318, *P* < 0.01) and WMH (*r* = −0.400, *P* < 0.01), but not global atrophy, age, or sex, were significantly correlated with the global FiCD value. CSVD patients with normal cognition showed decreased FiCD value mainly in the prefrontal areas (*P* < 0.01 with Monte Carlo correction). Compared with CSVD patients with normal cognition, CSVD patients with MCI showed significantly decreased FiCD value in enlarged frontal and parietal areas (*P* < 0.01 with Monte Carlo correction). Inter-group comparisons showed regional enhanced impairment of connectivity density in CSVD patients with MCI in the left superior frontal gyrus, the left precuneus, and the orbital part of the right inferior frontal gyrus (*P* < 0.01 with Monte Carlo correction). Regional FiCD value of frontal and parietal areas was associated with the cognitive function (*P* < 0.01). In conclusion, cognitively normal CSVD patients already have disruptions of structural connectivity. The extent and intensity of connectivity disruptions in frontal and parietal areas may underlie the mechanism of cognitive impairment in CSVD. Fiber connectivity density measurements may be helpful for quantitative description of structural cortical connectivity.

## Introduction

White matter hyperintensity of presumed vascular origin is one of the major imaging features of CSVD (Wardlaw et al., 2013). These subcortical ischemic lesions are heterogenous in histopathology and may represent demyelination, axonal loss, gliosis, and arteriolosclerotic changes (Gouw et al., 2011; Schmidt et al., 2011). Evidences have shown that WMH is associated with vascular cognitive impairment, particularly subcortical vascular MCI and subcortical vascular dementia (O’Brien et al., 2003). But the exact mechanisms of how WMH affects cognitive function remain largely unknown. In recent years, graph analyses using DTI have found associations between the disruptions of structural network connectivity and the cognitive impairment in CSVD patients (Lawrence et al., 2014; Tuladhar et al., 2016). More recently, it is found that the preferential disruptions of cortical connectivity in central brain areas may contribute to the development of cognitive impairment in CSVD patients (Tuladhar et al., 2017). All these evidences highlight the role of the disrupted structural connectivity in the pathophysiology of CSVD related cognitive impairment.

White matter hyperintensity is common in aging population (Breteler et al., 1994). It is not only observed in elderly with MCI and dementia, but also in cognitively normal elderly (Gootjes et al., 2004). Actually, over 10% of asymptomatic elderly people have confluent WMH on MRI (O’Sullivan, 2008). But it is yet not clear whether there are potential disruptions of structural connectivity associated with WMH in CSVD patients with normal cognition. If yes, it would raise another question that in face of disruptions of structural connectivity, why some CSVD patients remain cognitive normal while others do not. Comparisons of the connectivity characteristics between CSVD patients with normal cognition and CSVD patients with MCI may provide some clues.

In graph analyses, the entire cerebral cortex is typically parcelated into dozens of anatomical areas which are defined as network nodes (Tzourio-Mazoyer et al., 2002). Although it is a well-established parcelation scheme, the most optimal parcelation scale remains to be determined. Actually, it has been found that finer parcelation scale would better quantify the topological properties (Zalesky et al., 2010). Previous studies have found that cortical connectivity density could be reliably and accurately described with a more sophisticated parcelation scheme and further compared between groups in vertex levels (Liu et al., 2016, 2017). Thus, the purpose of this study was to investigate the potential disruptions of structural connectivity in CSVD patients with normal cognition and CSVD patients with MCI.

## Materials and Methods

### Participants

This study was approved by the Institutional Review Board of the hospital and informed consents were obtained from all subjects. All subjects underwent a comprehensive clinical evaluation that includes medical history, neurologic examinations, appropriate laboratory tests, and neuropsychological tests. CSVD patients who have WMH on MRI with or without lacunar infarcts were enrolled into the study consecutively. WMH were defined as diffuse moderate and severe confluent hyperintensities within WM on FLAIR images. Lacunar infarcts were defined as focal hyperintensities on T2-weighted images, 3 to 15 mm in size, and with corresponding hypointensities on T1-weighted images. Subjects with non-lacunar infarcts, cerebral hemorrhages, specific causes of WMH (e.g., multiple sclerosis), alcoholic encephalopathy were excluded. Based on the neuropsychological tests, CSVD patients with normal cognition were then classified as CSVD-NC group and CSVD patients with MCI were classified as CSVD-MCI group. MCI was defined as smaller than −1.5 standard deviation of local population norm in at least one cognitive domain in the standard neuropsychological assessments and do not meet the Diagnostic and Statistical Manual of Mental Disorders, Fourth Edition criteria for dementia. A control group of normal aging who have normal cerebral MRI and no cognitive impairment was setup. For all three groups, the exclusion criteria were: (1) age < 55 years; (2) severe systemic diseases (e.g., hepatitis), neurodegenerative diseases (e.g., Alzheimer’s disease), and severe psychiatric diseases (e.g., depression); (3) subjects without formal liberal education to ensure the accuracy of neuropsychological assessments; (4) any physical disorders that could lead to abnormal cognitive performance (e.g., prominent vision or hearing impairment); (5) subjects with transient ischemic attack within 3 months or lacunar infarcts observed as hyperintensities on DWI images were also excluded to avoid any acute effects on neuropsychological assessments. From May 2014 to June 2015, 25 patients were enrolled into the CSVD-MCI group and 35 patients were enrolled into the CSVD-NC group. Three CSVD-MCI patients and one CSVD-NC patient were excluded because of image artifacts on structural or diffusion images. Finally, a total of 22 CSVD-MCI patients, 34 CSVD-NC patients, and 35 controls were enrolled. The three groups were age, gender, and education matched.

### Neuropsychological Assessments

A battery of neuropsychological assessments involving the domains of executive, memory, language, and visuospatial functions was performed with all subjects. In particular, AVLT-immediate and AVLT-delayed were performed for memory assessment (Guo et al., 2007b), VFT-animal for language assessment (Guo et al., 2007a), CDT for visuospatial assessment (Shulman, 2000), Trail Making Test-part A (TMT-A) and -part B (TMT-B) for executive assessment (Lu and Bigler, 2002). In addition, MMSE as a common neurological assessment was also performed (Katzman et al., 1988). All scores were z-transformed based on raw data of all subjects to allow direct comparisons of performances between tests.

### MRI Acquisition

All subjects underwent MRI scan on a 3.0 Tesla scanner (GE healthcare, MR750) with a 32-channel head coil. DTI data were obtained in the axial plane using a single-shot echo-planar imaging sequence with the following parameters: TR/TE = 7500/66.2 ms, FOV = 256 mm × 256 mm, matrix = 128 × 128, flip angle = 90°, slice thickness = 2 mm, inter-slice spacing = 2 mm, number of diffusion gradient directions = 64, b-value = 1000 s/mm^2^, and scan time = 10 min 38 s.

Sagittal T1-weighted imaging used a 3D volumetric sequence with TR/TE/TI = 8.2/3.2/450 ms, FOV = 256 mm × 256 mm, matrix = 256 × 256, NEX = 1, flip angle = 12°, slice thickness = 1 mm, inter-slice spacing = 1 mm, scan time = 4 min 22 s.

Diffusion weighted imaging sequence also used a single-shot echo-planar imaging sequence with the TR/TE = 3000/98 ms, matrix = 160 × 160, b-value = 1000 s/mm^2^, and scan time = 42 s. T2-weighted fast spin echo images were scanned with the TR/TE = 4599/102 ms, matrix = 320 × 224, NEX = 2 and T2 FLAIR were scanned with the TR/TE/TI = 8400/160/2100 ms, matrix = 256 × 256, NEX = 1. The slice thickness, inter-slice spacing, and FOV were 5 mm, 1.5 mm, and 240 mm × 240 mm, respectively.

### Data Analysis

#### MRI Markers of CSVD

The following MRI markers of CSVD were analyzed. To quantitatively assess the WMH volume, we used a fast and robust automated quantification method as described previously (Shi et al., 2013). Lacunar infarcts were manually identified by two experienced observers with the open source software ITK-SNAP (Penn Image Computing and Science Laboratory) (Yushkevich et al., 2006). Brain atrophy was evaluated with an automatic quantification program (Wang et al., 2019). Relative brain volume was calculated as the absolute brain volume divided by the intracranial volume to avoid any potential effects of brain size variations.

#### Preprocessing

First, the cortical GM–WM interface was constructed using Freesurfer software (Fischl, 2012) based on 3D T1-weighted images. The major steps included intensity normalization, non-brain tissue removal, automated Talairach transformation, GM–WM segmentation (Fischl et al., 2002), tessellation of GM–WM boundary, automated topology correction (Segonne et al., 2007), and intensity gradients based surface deformation which allowed optimal placement of tissue borders (Fischl and Dale, 2000). Second, diffusion data was preprocessed using the FSL toolbox (Jenkinson et al., 2012). This included head motion correction, eddy currents correction, and brain extraction in DWI. Third, the 3D T1-weighted images and the diffusion images were co-registered with the constructed GM–WM interface for each subject.

#### Cortical Connectivity Analysis

**FIGURE 1:**
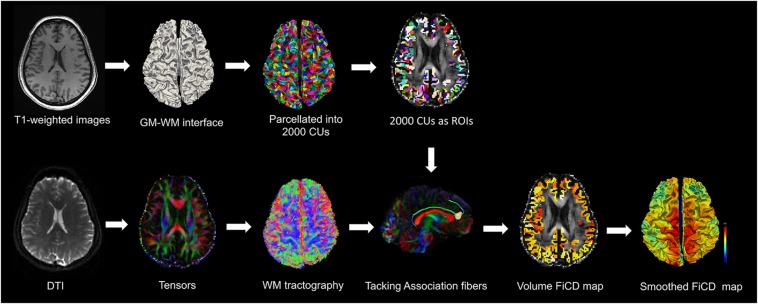
The overall workflow of FiCD mapping. First, the cortical GM–WM interface is extracted from high spatial resolution T1-weighted images and is then parcelated into 2000 CUs. Second, after WM tractography with DTI images, the CUs are transformed into the tractography space. Third, the CUs are used as ROIs to select association fibers and then FiCD value is calculated for each CU. Fourth, the generated whole-cortex FiCD map is projected onto the cortical surface and then registered to common brain surface. After Gaussian smoothing, the FiCD map is ready for group-wise comparisons.

The whole-cortex structural connectivity was analyzed with the inhouse developed algorithm named “FiCD mapping” (Liu et al., 2016, 2017). By combining cortical surface reconstruction and diffusion tractography technique, FiCD mapping is able to match fiber bundles to the reconstructed cortical surface (Liu et al., 2016, 2017). The overall workflow of FiCD mapping is illustrated in Figure 1. In brief, the GM–WM interface of the whole cortex was parcelated into 2000 CUs using the *k*-medois algorithm (Kaufman and Rousseeuw, 1987). The CUs in T1-weighted images were transformed into the tractography space and used as region of interests for whole brain deterministic fiber tracking. In DSI Studio, the generated streamlines associated with a CU was named association fibers of the CU. Tracking was terminated at factional anisotropy (FA) threshold = 0.14, turning angle threshold = 45°, and fiber length constraint of 30–300 mm. The FiCD value of a CU was calculated according to the following equation:

$$\mathrm{FiCD}=\frac{\left(M\,A_{f\,1}+M\,A_{f\,2}+\cdots +M\,A_{f\,N}\right)}{V_{C\,U}}$$

Where *f* represents a generic streamline of the association fiber set of a CU; *N* refers to the total number of the generated association fibers connecting to the CU; *MA* represents the mean anisotropy value; *V*_*CU*_ refers to the volume of the CU. The FiCD value of a CU represents the connectivity density of the cortical region as it incorporates the information of fiber strength (mean FA value) and degree (fiber count) which was divided by the CU volume to correct for the inhomogeneity of CU size. The FiCD values of all CUs of the whole cortex were used to construct the whole cortical FiCD map. For intergroup vertex-wise comparisons, the FiCD maps of all subjects within a group were spatially normalized to the template brain surface (Fischl et al., 1999) using non-linear registration and then smoothed using a Gaussian kernel with full width at half maximum = 10 mm to reduce noise. Finally, the FiCD value of the template brain surface between groups were compared using a General Linear Model with the Qdec module of Freesurfer (Fischl, 2012). Statistical significance was thresholded at *P* < 0.01 with a Monte Carlo null-Z simulation to correct for multiple comparisons with 10000 iterations. In addition, regional FiCD values were calculated by averaging the FiCD values of all the CUs within the ROIs which were selected from the regions with statistical significance in group-wise comparisons.

### Statistical Analysis

Statistical analysis was performed using SPSS software (Version 20.0, IBM) with regards to data distribution. One-way ANOVA, Chi-square test, and Kruskal–Wallis H test were used for group comparisons of the demographics and the neuropsychological tests. WMH volume was log transformed because of its skewed distribution. Student’s *t*-test was used for comparisons of WMH volume and lacunar infarct volume between the CSVD-MCI group and the CSVD-NC group. To quantitatively reveal the severity of the impairment of the global connectivity of the CSVD-MCI group and the CSVD-NC group compared with that of the control group, reduction of the mean global FiCD value of the two groups were divided by the mean global FiCD value of the control group respectively to get the reduction ratio. The relative brain volume and the global FiCD value between the three groups were compared using One-way ANOVA with Bonferroni test and Tamhane’s T2 test for *post hoc* tests respectively. The correlation between global FiCD value and the lacunar infarct volume, the WMHs volume, brain atrophy was performed with linear regression analysis. Pearson correlation was performed between regional FiCD value and the cognitive function after controlling for age, sex, and education. Statistical significance was defined at *P* < 0.05.

## Results

### Lesion Load and Distribution

The baseline characteristics of CSVD patients were summarized in Table 1. Of the conventional risk factors of cerebrovascular disease, CSVD patients, irrespective of the cognitive function, had significantly higher prevalence of hypertension than the controls (*P* = 0.001).

**TABLE 1:** Group demographics and clinical characteristics.

	Control	CSVD-NC	CSVD-MCI	
	*n* = 35	*n* = 34	*n* = 22	*P*-value
**General characteristics**				
Age, y	62.9 ± 6.9	64.6 ± 6.6	65.1 ± 6.6	0.437
Sex, male (%)	20 (57)	22 (65)	14 (64)	0.790
Education*	9 ± 3	9 ± 4	9 ± 6	0.268
**Vascular risk factors**				
Hypertension, n (%)	9 (26)	23 (68)^†^	12 (59)^‡^	0.001
Diabetes mellitus, n (%)	4 (11)	6 (18)	2 (9)	0.604
Dyslipidemia, n (%)	6 (17)	9 (26)	5 (23)	0.643
Smoking, n (%)	10 (29)	14 (41)	11 (50)	0.248
Alcohol, n (%)	6 (17)	4 (12)	4 (18)	0.757
**Neuropsychological**				
**assessments**				
MMSE	29 (0.9)	29 (1.2)	26 (3.0)^‡§^	<0.001
TMT-A	59.2 (25.1)	59.5 (20.2)	134.3 (62.9)^‡§^	<0.001
TMT-B	113.8 (38.1)	127.4 (39.9)	248.6 (74.7)^‡§^	<0.001
VFT	23.5 (4.8)	22.4 (4.8)	16.6 (6.0)^‡§^	<0.001
AVLT-immediate	7.7 (1.8)	6.6 (2.5)	4.6 (2.4)^‡§^	<0.001
AVLT-delayed	7.5 (1.8)	6.2 (2.6)	4.0 (2.7)^‡§^	<0.001
CDT*	4 (0)	4 (0)	3 (3)^‡§^	<0.001

*Median ± interquartile range. ^†^Indicates significant differences between CSVD-NC group and control group. ^‡^Indicates significant differences between CSVD-MCI group and control group. ^§^Indicates significant differences between CSVD-MCI group and CSVD-NC group. MMSE, Mini-Mental State Examination; TMT-A and -B, Trail Making Test part A and part B; VFT, Verbal Fluency Test; AVLT- immediate and -delayed, Auditory Verbal Learning Test immediate and delayed recall; CDT, Clock Drawing Test.

**FIGURE 2:**
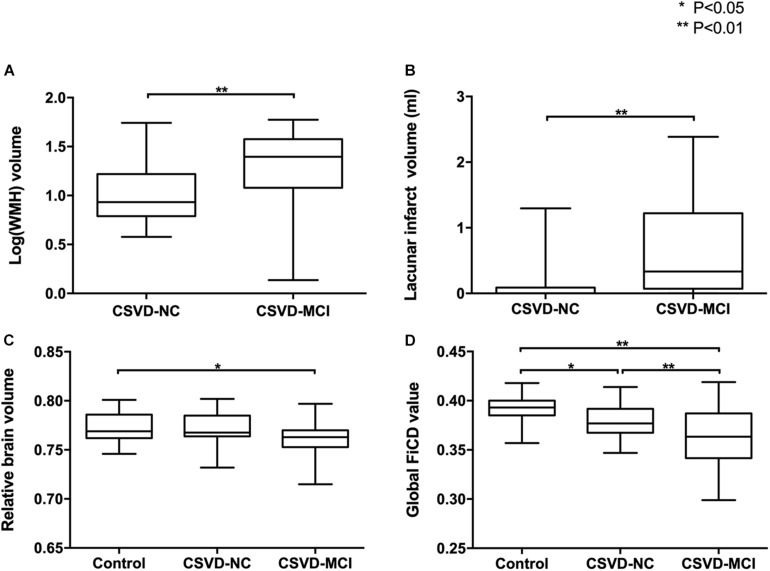
Group-wise comparisons of WMH, lacunar infarcts, brain atrophy, and global FiCD value. The volumes of WMH **(A)** and lacunar infarcts **(B)** of the CSVD-MCI group are significantly larger than those of the CSVD-NC group (*P* < 0.01). The relative brain volume **(C)** is significantly decreased in the CSVD-MCI group (*P* < 0.05) but not in the CSVD-NC group (*P* > 0.05) compared with the controls. The global FiCD value **(D)** is significantly decreased in the CSVD-NC group (*P* < 0.05) and CSVD-MCI group (*P* < 0.01) than in the controls.

**FIGURE 3:**
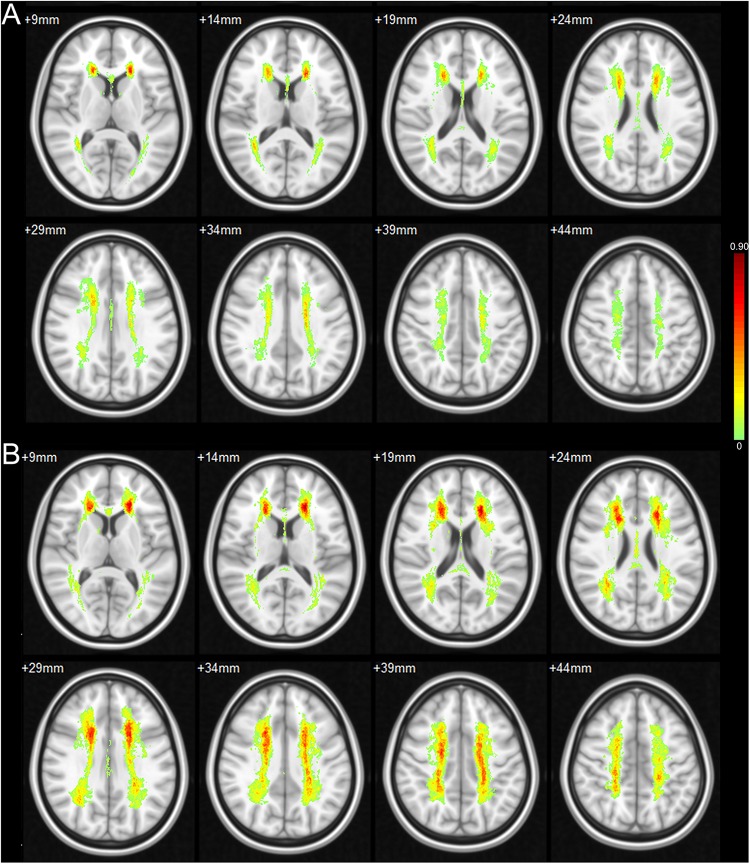
Possibility map of WMH in CSVD-NC group and CSVD-MCI group. The WMH are mainly distributed in the periventricular WM, deep WM of bilateral frontal and parietal lobes in both the CSVD-NC group **(A)** and the CSVD-MCI group **(B)**. Compared with the CSVD-NC group, the lesion load of WMH is increased in the frontal and parietal system in the CSVD-MCI group.

**FIGURE 4:**
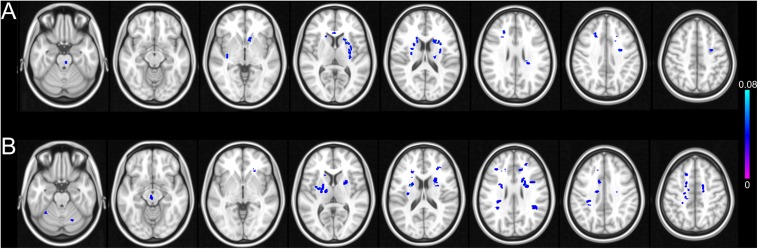
Possibility map of lacunar infarcts in CSVD-NC group and CSVD-MCI group. The lacunar infarcts are mainly distributed in the bilateral basal ganglia, deep WM of frontal and parietal lobes in both the CSVD-NC group **(A)** and the CSVD-MCI group **(B)**. Compared with the CSVD-NC group, the lesion load of lacunar infarcts is increased in the frontal and parietal system in the CSVD-MCI group.

It is interesting to note that, though the CSVD patients were enrolled into the study successively and then classified into the CSVD-NC group and CSVD-MCI group based on the cognitive assessments, the volumes of lacunar infarcts and WMH of the CSVD-MCI group were significantly larger than that of the CSVD-NC group (Figures 2A,B). The distributions of the WMH and lacunar infarcts of the CSVD patients were shown in Figures 3, 4 respectively. In both the CSVD-NC group and the CSVD-MCI group, the distribution of WMH and lacunar infarcts was similar. But the lesion load of WMH and lacunar infarcts was increased in the frontal and parietal system in the CSVD-MCI group compared with the CSVD-NC group. The relative brain volume of the CSVD-MCI group was significantly smaller than that of the control group (*P* < 0.05) (Figure 2C).

### Global Fiber Connectivity Density

**FIGURE 5:**
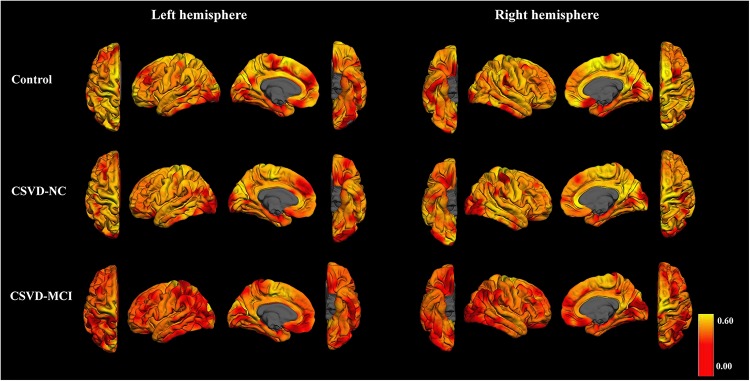
FiCD maps of control group, CSVD-NC group, and CSVD-MCI group. In the control group and CSVD-NC group, bilateral medial frontal, and parietal areas show the highest FiCD value. The CSVD-MCI group shows dramatic decrease of FiCD value in bilateral frontal and parietal areas.

Figure 5 shows that in the control group and CSVD-NC group, areas with the highest FiCD value include medial frontal and parietal areas of both hemispheres, indicating the highest connectivity density in these areas. In the CSVD-MCI group, bilateral paracentral areas were still with the highest FiCD value and bilateral frontal and parietal areas were with the lowest FiCD value. Quantitatively, compared with the control group, the CSVD-MCI patients showed 7.91% (*P* < 0.01) and the CSVD-NC patients showed 3.06% (*P* < 0.05) reduction of the global FiCD value (Figure 2D). In the regression analysis of the global FiCD value, only the lacunar infarct volume (*r* = −0.318, *P* < 0.01) and the WMH volume (*r* = −0.400, *P* < 0.01), but not the relative brain volume, age, or sex, were significantly correlated with the global FiCD values, indicating involvement of lacunar infarcts and WMH in the impairment of structural connectivity of CSVD patients.

### Vertex-Wise Intergroup Comparison of Fiber Connectivity Density

**FIGURE 6:**
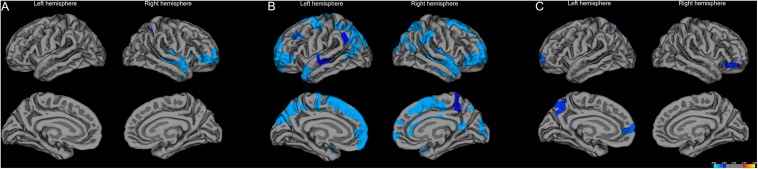
Vertex-wise comparisons of FiCD mapping between control group, CSVD-NC group, and CSVD-MCI group. **(A)** Compared with the control group, the FiCD of the CSVD-NC group is significantly decreased in the right prefrontal areas and right superior temporal gyrus. **(B)** The FiCD of the CSVD-MCI group is decreased in bilateral frontal and parietal areas relative to the controls. **(C)** Compared with the CSVD-NC group, the FiCD of the CSVD-MCI group is decreased in the anteromedial and dorsolateral part of the left superior frontal cortex, the left precuneus, and the orbital part of the right inferior cortex. *P* < 0.01 with Monte Carlo correction for all comparisons.

Compared with the control group, the CSVD-NC group showed significantly reduced FiCD mainly in the prefrontal cortex, including the right superior frontal gyrus, the right rostral middle frontal gyrus, the pars triangularis of the right inferior frontal gyrus (*P* < 0.01 with Monte Carlo correction) (Figure 6A and Table 2). The FiCD was also reduced in the right superior parietal gyrus, the right superior and middle temporal gyrus, and the left insular in the CSVD-NC group (*P* < 0.01 with Monte Carlo correction). The CSVD-MCI group showed reduced FiCD in enlarged brain areas which extended from frontal areas posteriorly to parietal areas, including bilateral superior frontal gyrus and the medial and lateral parietal regions of both hemispheres (*P* < 0.01 with Monte Carlo correction) (Figure 6B and Table 3). Moreover, compared with the CSVD-NC group, the CSVD-MCI group showed further decreased FiCD in the left superior frontal cortex, the left precuneus, and the orbital part of the right inferior frontal gyrus (*P* < 0.01 with Monte Carlo correction) (Figure 6C and Table 4), indicating selectively enhanced impairment of structural connectivity to these areas in CSVD patients with MCI.

**TABLE 2:** Regions showing statistical differences in FiCD mapping between CSVD-NC group and control group.

Cluster	Size				
number	(mm^2^)	Peak tal coordinate			Annotations
		X	Y	Z	
1	601.88	–36.1	–20.8	–3	Left insula
2	2721.34	46.4	–33.2	–4	Right middle temporal gyrus
3	1916.15	40.2	38.2	0.6	Right pars triangularis
4	753.5	35.4	–51.8	38.4	Right inferior parietal gyrus
					

**TABLE 3:** Regions showing statistical differences in FiCD mapping between CSVD-MCI group and control group.

Cluster	Size				
number	(mm^2^)	Peak tal coordinate			Annotations
		X	Y	Z	
1	6912.33	–7.3	15.7	58	Left superior frontal gyrus
2	993.12	–36.4	29.4	28.1	Left rostral middle frontal gyrus
3	8909.31	–13.5	–42.3	47.3	Left precuneus
4	697.93	–43.2	–48.8	18.8	Left inferior parietal gyrus
5	1896.01	–35.9	–21.3	–2.3	Left insula
6	704.54	–54.8	–13.4	–5.9	Left superior temporal gyrus
7	7175.36	22	57.2	11.8	Right rostral middle frontal gyrus
8	1496.18	14.4	30.2	21.3	Right superior frontal gyrus
9	708.42	14.8	–38.3	57.5	Right precuneus
10	3669.04	25.7	–52.2	46.6	Right superior parietal gyrus
11	2039.92	51	–44.1	22.8	Right inferior parietal gyrus
12	4644.02	48.4	11.5	–18.5	Right superior temporal gyrus
13	1263.74	15.1	–86.8	7.9	Right pericalcarine

**TABLE 4:** Regions showing statistical differences in FiCD mapping between CSVD-MCI group and CSVD-NC group.

Cluster	Size				
number	(mm^2^)	Peak tal coordinate			Annotations
		X	Y	Z	
1	937.89	–13.8	48.3	0.4	Left superior frontal gyrus
2	815.58	–8.6	–59.1	48.9	Left precuneus
3	746.44	46.1	37.4	–12.1	Right orbital part of inferior
					frontal gyrus
					

### Focal Connectivity Density and Cognitive Function

Since the FiCD of the CSVD-MCI group was further decreased in regions of the left superior frontal cortex, the left precuneus, and the orbital part of the right inferior frontal cortex than that of the CSVD-NC group, correlation of regional FiCD value with the cognitive function was further evaluated in these areas. The FiCD value of the left superior frontal cortex, the left precuneus, and the orbital part of the right inferior frontal cortex correlated significantly with the cognitive function after controlling for age, sex, and education (Table 5).

**TABLE 5:** Correlation between regional FiCD value and cognitive function after controlling for age, sex, and education.

	Left superior		Left		Right inferior	
	frontal cortex		precuneus		frontal cortex	
	*r*	*P*	*r*	*P*	*r*	*P*
MMSE	0.460	0.001	0.480	0.000	0.305	0.027
Memory	0.382	0.005	0.413	0.002	0.223	0.108
Language	0.397	0.003	0.415	0.002	0.232	0.095
Visuospatial	0.568	0.000	0.512	0.000	0.151	0.279
Executive	–0.614	0.000	–0.711	0.000	–0.546	0.000

*MMSE, Mini-Mental State Examination.*

## Disscusion

The current study specifies the different characteristics of the connectivity impairment in CSVD patients with normal cognition and CSVD patients with MCI. We found that CSVD patients with normal cognition had impairment of structural connectivity mainly in the prefrontal cortex. And CSVD patients with MCI showed more prominent connectivity impairment in enlarged brain areas which extended from frontal areas posteriorly to parietal areas. Moreover, the inter-group comparisons showed that CSVD patients with MCI had selectively enhanced impairment of structural connectivity in the left superior frontal gyrus, the left precuneus, and the orbital part of right inferior frontal gyrus than the CSVD patients with normal cognition.

Cerebral small-vessel disease lesions are rather heterogenous in terms of histopathology (Gouw et al., 2011; Schmidt et al., 2011). For example, pathological substrates of WMH range from degeneration of aging to incomplete infarct associated with ischemia (Schmidt et al., 2011). These histopathological changes may bring different effects on cognitive function (Gouw et al., 2011). We found that CSVD patients with MCI have similar but more extended distributions of CSVD lesions than CSVD patients with normal cognition. Quantitatively, CSVD patients with MCI have higher global load of CSVD lesions and correspondingly severer reduction of global FiCD than the CSVD patients with normal cognition. These observations support the involvement of CSVD lesions in the impairment of structural connectivity and cognitive function (Lawrence et al., 2014; Tuladhar et al., 2016, 2017). Moreover, CSVD patients with MCI have the shared vascular risk factor of hypertension with cognitively normal CSVD patients. It suggests a common arteriolosclerosis basis in CSVD patients with and without cognitive impairment. Hypertension may cause thickening and hardening of arteriole wall and narrowing of arteriole lumen, which may reduce cerebral blood flow and result in cerebral hypoperfusion (Novak et al., 2003). Cerebral hypoperfusion reduces supplies of blood oxygen and nutrients to brain parenchyma, which may cause neurodegenerative changes and subsequent cognitive impairment (Novak et al., 2003; Muller et al., 2010).

The prefrontal cortex plays an important role in the “top–down” processing and cognitive control (Miller and Cohen, 2001). Patients with traumatic or vascular prefrontal damage show significant deficits in executive behaviors (Leclercq et al., 2000). In the current study, albeit reduced FiCD in prefrontal cortex, some CSVD patients remained cognitively normal. And cognitively normal elderly has been found to have GM volume loss associated with WMH predominantly in the frontal cortex (Raji et al., 2012). These evidences lend supports to the existence of brain reserve which refers to the ability of adult brains to endure neuropathological processes and minimize clinical symptoms (Stern, 2002). From an anatomical perspective, brain reserve may help to prolong the onset of cognitive symptoms in CSVD until pathological damages reach a threshold (Stern, 2002). It may be hard to define the exact threshold of pathological damages between normal cognition and cognitive impairment in CSVD. By deliberate comparisons between CSVD patients with normal cognition and CSVD patients with MCI, our study may provide some valuable information. Quantitatively, CSVD patients with MCI have severer reduction of global connectivity density than CSVD patients with normal cognition. Topologically, CSVD patients with MCI have more extended and selectively enhanced impairment of connectivity density in fronto-parietal areas than CSVD patients with normal cognition. And these changes of connectivity density are consistent with the lesion load and distribution of WMH and lacunar infarcts between the two groups. These results indicate that the extent and intensity of connectivity impairment in frontal and parietal areas may be crucial for the pathophysiology of CSVD related cognitive impairment.

Previous studies have found that disruptions of structural connectivity to central network nodes may play an important role in the cognitive impairment of CSVD (Lawrence et al., 2014; Tuladhar et al., 2016). These central network nodes include superior frontal, superior parietal, precuneus, and subcortical regions (Gong et al., 2009; van den Heuvel and Sporns, 2011). Compared with non-central nodes, they are more densely connected with distributed brain areas (Gong et al., 2009; van den Heuvel and Sporns, 2011). And these nodes may play a more important role in brain functions, particularly information integration due to their central locations (Gong et al., 2009; van den Heuvel and Sporns, 2011). For example, the prefrontal cortex connects with virtually all brain areas that serve sensory, motor, and other functions (Miller and Cohen, 2001). We found that compared with CSVD patients with normal cognition, CSVD patients with MCI have extended and regional enhanced impairment of structural connectivity in these central areas, mainly in the frontal and parietal areas. Moreover, regional connectivity of the frontal and parietal areas was associated with the cognitive function. These evidences indicate that in the CSVD patients with MCI, the extended and enhanced impairment of structural connectivity to these central areas may have reduced the abilities of the brains to exploit strengthened utilization of brain networks or recruit alternative networks to maintain normal cognition (Stern, 2002). These evidences also support that the connectivity impairment to frontal and parietal areas may contribute to the failure of cognitive reserve in CSVD patients.

The accuracy of the estimates of regional structural connectivity has been found to be associated with the parcelation scale applied (Zalesky et al., 2010). For example, a network analysis with a coarse parcelation may fail to characterize some branch of a forking U-fiber. By parcelating the whole cortex into 2000 CUs, the current study was able to delicately describe the connectivity status throughout the brain. Based on the vertex-wise statistical comparisons among groups, it is possible to show the regional reduction of FiCD of CSVD patients relative to controls. To some extent, the FiCD value of a CU actually reflects the density and integrity of association fibers as it considered both the number of all association fibers connected to a CU and the weighted anisotropy value of each associated fiber. In addition, FiCD mapping has been found to be able to accurately and reliably tract fibers and locate the affected cortical regions in post-stroke patients (Liu et al., 2017). These evidences indicate that FiCD mapping may be helpful for our understanding of structural network changes in CSVD.

There are certain limitations in our study. First, the relative small sample size and limited number of neuropsychological tests were certain drawbacks. Second, although deterministic tractography was a conventional method for estimation of structural brain network, it has limitations such as failure in complex WM and low signal-to-noise ratio. Further improvements on diffusion imaging and tractography algorithms can address these issues. Third, the current study did not enroll CSVD patients with dementia. Thus, the characteristics of the connectivity disruptions need to be further described in those patients. Fourth, cerebral microbleed was not investigated. Cerebral microbleeds occur most commonly in the cerebral cortex while the cortical network connectivity was analyzed based on the investigation of the diffusion tensor of WM fibers. Thus, the cortical network connectivity was minimally affected by cortical microbleeds.

## Conclusion

Cognitively normal CSVD patients already have disruptions of structural connectivity. The extent and intensity of connectivity disruptions in frontal and parietal areas may underlie the pathophysiological mechanisms of CSVD associated cognitive impairment.

## Data Availability Statement

All datasets generated for this study are included in the article/supplementary material.

## Ethics Statement

The studies involving human participants were reviewed and approved by the Institutional Review Board of Tongji Hospital of Tongji Medical College of Huazhong University of Science and Technology. The patients/participants provided their written informed consent to participate in this study.

## Author Contributions

All authors: guarantor of integrity of the entire study, experimental studies/data analysis, manuscript preparation and editing, and final approval. WzZ, CL, LS, DW, and YQ: study concepts and design. WzZ, CL, LS, DW, PS, SY, YQ, and WhZ: literature research. WzZ, CL, ZW, SZ, YQ, WhZ, SY, and XT: clinical studies. CL, LS, DW, PS, YQ, and WzZ: statistical analysis.

## Conflict of Interest

The authors declare that the research was conducted in the absence of any commercial or financial relationships that could be construed as a potential conflict of interest.

## Abbreviations

AVLT: Auditory Verbal Learning Test
CDT: Clock Drawing Test
CSVD: cerebral small-vessel disease
CUs: cortical units
DTI: diffusion tensor imaging
DWI: diffusion weighted imaging
FA: fractional anisotropy
FiCD: fiber connectivity density
FLAIR: fluid attenuated inversion recovery
FOV: field of view
GM: gray matter
MCI: mild cognitive impairment
MMSE: mini-mental state examination
MRI: magnetic resonance imaging
TE: echo time
TI: time interval
TMT: Trail Making Test
TR: repetition time
VFT: Verbal Fluency Test
WM: white matter
WMH: white matter hyperintensity.
